# An Epidemiological Investigation of Inter-Developmental, Biopsychosocial Impairment among Children and Adolescents in Foster Care

**DOI:** 10.1007/s40653-025-00692-3

**Published:** 2025-02-05

**Authors:** Michael Tarren-Sweeney, Kenneth Patrick Nunn

**Affiliations:** 1 Child and Family Psychology department, Canterbury University, Canterbury, New Zealand; 2 Department of Psychological Medicine, Elver Trauma Service DCJ and SWSLHD, Children’s Hospital at Westmead, University of Sydney (Westmead Campus), Sydney, Australia

**Keywords:** Out-of-home care, Foster care, Developmental psychopathology, Complex symptomatology, Assessment Checklist for Children, Assessment Checklist for Adolescents, Inter-developmental impairment, Speech and language difficulties, Intellectual disability

## Abstract

Early exposure to chronic maltreatment and other biopsychosocial adversities accounts for high prevalence of developmental impairments among children residing in foster care and other types of statutory out-of-home care (OOHC), including: complex, trait-like mental health difficulties; intellectual disability (ID); and speech and language difficulties (SLD). However, little is known about the characteristics and prevalence of co-occurring mental health difficulties and developmental disabilities among this population – which we refer to as *inter-developmental impairment.* The present article reports findings from epidemiological surveys of pre-adolescent school-aged children (*N* = 347) and adolescents (*N* = 230) residing in foster and kinship care in New South Wales, Australia. Mental health was measured from caregiver-reported Child Behavior Checklist and Assessment Checklist for Children / Assessment Checklist for Adolescents scores; and ID and SLD were reported in a caregiver questionnaire. The proportions of children and adolescents with caregiver-reported ID were 22.5% (78/347) and 30.0% (69/230) respectively, while the proportions with SLD were 21.6% (75/347) and 15.7% (36/230) respectively. While mental health case rates were high among the aggregate child (67%) and adolescent (60%) samples, those with ID and/or SLD were considerably more likely to have clinical-level mental health difficulties compared to those without ID or SLD, with odds ratios ranging from 1.9 to 6.5. The prevalence of inter-developmental impairment (defined as having ID *and/or* SLD, as well as mental health caseness) among the child and adolescent samples was 23.9% and 28.7% respectively. Children and adolescents with inter-developmental impairment on average had with more complex symptomatology than did mental health cases without reported developmental difficulties. The article discusses mechanisms accounting for inter-developmental impairment among formerly maltreated children residing in foster care, and implications for clinical services.

## Introduction

Children and adolescents residing in long-term statutory out-of-home care (OOHC, including family-based foster care and kinship care, as well as residential care) constitute the most identifiably disadvantaged child population in the developed world. Estimates of the distribution of developmental impairments in this population are remarkably consistent. Population surveys and cohort studies have consistently found that children and young people in OOHC have high mean levels and rates of mental health difficulties. Though rates vary a little by survey and location, up to half of such children have clinical-level mental health difficulties, and another 20–25% have difficulties approaching clinical significance (Engler et al., 2022; Oswald et al., 2010). Furthermore, many children in OOHC manifest complex mental health difficulties that are not well conceptualized within standard diagnostic classifications (DeJong, 2010; Tarren-Sweeney, 2021). These include combinations of: dysregulated affect and behaviour; inattention / over-activity; trauma-related anxiety, hypervigilance and re-experiencing trauma; dissociation and sensory disturbances; negative and unstable perceptions of self and others; attachment disorders and other attachment-related interpersonal difficulties; conduct problems, oppositional-defiance, low empathy and aggression; sexual behaviour problems; food maintenance behaviours (hoarding, storing, gorging); restricted, odd and stereotypic behaviours; depression; self-injury; and suicidal ideation and behaviour (Tarren-Sweeney, 2021).

The cognitive and language functioning of this population have been less thoroughly studied than their mental health. Furthermore, interpreting cognitive and language impairment rates for children placed in residential care is complicated by the fact that in some jurisdictions residential OOHC serves multiple populations, including children placed in care due to them having a disability rather than being maltreated – whereas in other jurisdictions, OOHC residential care is separate from disability residential care (McConkey et al., 2014). However, a modest number of studies of children in foster care have identified high rates of intellectual and speech / language impairment. A recent population study of the development of school-aged Swedish foster children (aged 3–9 years) measured a mean full-scale Wechsler IQ of 90.7, which is 9 standard points below the general population mean (Tordön et al., 2020). Equivalent mean IQ scores measured among two US samples of children entering foster care were 88.6 (*N* = 120, ages 6–17 years) (Viezel et al., 2015) and 88.0 (*N* = 3144, age 6 and above) (Evans, 2001). These means reflect sizeable variation from normative development (15 IQ points equates to one standard deviation). A US study that screened developmental functioning of maltreated young children (0–6 years, *N* = 205) prior to entering foster care measured 30% as having a ‘significant’ developmental delay on the Bayley-II mental development index (Leslie et al., 2002), as compared to 1.5% in the general population. It is expected that measured impairment is greatest at the point of entry into care, given that many children experience some developmental ‘catch-up’ after being placed in OOHC. Consequently, cross-sectional studies of children in family-based foster care have measured slightly lower rates of intellectual disability (ID) within the range of 13–24%, as compared to around 2% of children in the general population (13.7%, *N* = 8830, 0–17 years (Heinrichs et al., 2018); 16.4%, *N* = 125, 0–3 years (Klee et al., 1997); 22.5%, *N* = 347, 4–11 years (Tarren-Sweeney, 2008); 24.2%, *N* = 149, 0–18 years (Hochstadt et al., 1987).

Comparable mean levels of speech and language difficulties (SLD) have also been identified among this population. A US nationally-representative cohort study (*N* = 963) of the development of maltreated infants included measures of expressive and receptive language skills from the first year of life through to ages 5–6 (Administration for Children Youth and Families, 2001). Mean age-standardized *expressive* language scores (normative mean = 100; standard deviation = 15) ranged over five study waves from 87 to 93, and 88 to 96 for children in foster care (*N* = 258) and kinship care (*N* = 158) respectively – while mean *receptive* language scores ranged from 87 to 95 and 87–96 respectively (Stacks et al., 2011). However, estimates of the rates of children in care with delayed language development vary widely from 18 to 73% (Hochstadt et al., 1987; Maguire et al., 2021; Stock & Fisher, 2006; Tarren-Sweeney, 2008), reflecting in large part different definitions of what constitutes ‘delayed’ language development.

### Inter-Developmental Impairment

Children at large with ID are two to four times more likely to have clinically significant mental health difficulties (psychiatric disorder) than other children (Brereton et al., 2006; Munir, 2016). Furthermore, children with co-occurring ID and autism spectrum disorder (ASD) experience even greater levels of comorbid psychopathology than do other children with ID (Brereton et al., 2006), with comorbid behaviour problems exerting a greater impact on parental and family functioning than children’s primary developmental difficulties (Herring et al., 2006).

While it is established that maltreated children placed in OOHC manifest high rates and levels of mental health, cognitive and language difficulties, most research has examined the phenomenology and functional implications of these different impairments in isolation from each other – while just a few studies have reported rates of co-occurring impairment. The longitudinal English-Romanian Adoption study found that adopted infants who were profoundly deprived in orphanages, subsequently manifested high rates of inter-developmental impairment through childhood (notably ASD symptoms, inattention / overactivity, attachment disorders, emotional problems and behavioural problems, and cognitive impairment) (Sonuga-Barke et al., 2017). A large epidemiological study of children in OOHC (*N* = 8830, ages 0–17), found that children with developmental delay were twice as likely as non-delayed children to have a mood or anxiety disorder (Heinrichs et al., 2018). Another study identified a relationship between social cognitive deficits and biases (theory of mind) and disinhibited attachment disorder behaviour among a small sample (*N* = 63) of adolescents in OOHC (Kay & Green, 2016).

Otherwise, little is known about the prevalence and impact of complex, *inter-developmental impairment* among this population. To our knowledge, no study has reported detailed inter-relationships or patterns of co-occurring mental health, relational, language and other developmental difficulties among children in care. Increasing our understanding of inter-developmental impairment has major implications for policy and developmental-clinical practice pertaining to this vulnerable population, as well as provision of developmental-clinical services. To address this gap, the present paper reports relationships between level and complexity of clinical mental health difficulties and caregiver-reported ID and SLD – as well as the relative impact these have on burden of care – among population samples of Australian children and adolescents in long-term foster and kinship care.

## Method

The Children in Care study (CICS) was a prospective epidemiological study of the mental health and developmental risk exposure of children in long-term foster care, in New South Wales (NSW), Australia. It consisted of a cross-sectional baseline survey of pre-adolescent children (ages 4–11) conducted between November 1999 and January 2003 (*N* = 347) (Tarren-Sweeney & Hazell, 2006); and a cross-sectional adolescent survey conducted between 2009 and 2011 (*N* = 230) (Tarren-Sweeney, 2018). The adolescent survey included 85 participants from the child survey, who constituted a 7- to 9-year prospective follow-up sample. At the time the study was conducted, children were only placed into long-term statutory OOHC in NSW because the Children’s Court determined they had an ongoing need for protective care (e.g. maltreatment and / or abandonment). The relevant legislation did not allow children to be placed in court-ordered long-term OOHC due to them having serious mental health or developmental difficulties.

A large number of demographic, developmental, mental health, and likely risk and protective factors were measured in the child and adolescent surveys, as well as prospectively for the follow-up participants, which have been detailed previously (Tarren-Sweeney & Hazell, 2006). Factors pertaining to children’s mental health, development, disabilities, education and present status (e.g. type and makeup of present placement, recent life events) were measured from carer questionnaires at both time points. Historical data (e.g. maltreatment history, care history, and birth family factors) were measured retrospectively from the state child welfare database at both time points. Since historical events were mostly recorded on the child welfare database shortly after they occurred, these data are thought to be more reliable than those typically obtained in a retrospective design. However, only a limited set of such data were recorded for the 147 newly-recruited adolescents. Children and young people were not directly involved in the study.

The study was approved by the Human Ethics Committees of the University of Newcastle in Australia (child and adolescent surveys), and the University of Canterbury in New Zealand (adolescent survey). All procedures performed in studies involving human participants were in accordance with the ethical standards of the institutional research committees and with the 1964 Helsinki declaration and its later amendments or comparable ethical standards.

### Participants

#### Pre-Adolescent Child Sample

The baseline sampling frame was defined as all 4–9 year-old children residing in foster or kinship care in New South Wales, Australia, under guardianship of the statutory child welfare agency. The study sought to enlist the carers of all eligible children, with one proviso: that their contact details could be confirmed via either the electoral roll or a telephone listing. The sampling frame consisted of 819 children, of whom 198 did not have confirmed contact details. Questionnaires were thus sent to the carers of 621 eligible children (the study sample), with 347 returns (56% response rate).

#### Adolescent Sample

##### Follow-up Participants

The adolescent survey attempted to obtain follow-up social care and developmental data for all participants in the baseline child survey who remained in foster or kinship care in early to mid-2009, seven to nine years after their baseline data were gathered. Of the 347 baseline survey participants, 231 remained in court-ordered OOHC at follow-up. Of these, 66 were residing in placements that did not have verifiable contact details, and whose carers could not be located by telephone. There were thus 165 young people that were eligible for inclusion in the follow-up survey, and could be reliably located. Of these, questionnaires were returned for 85 young people, representing a 51.5% response rate. However, these participants represent only 37% of baseline participants remaining in care.

##### Additional Adolescent Survey Participants

The sampling frame for recruiting additional adolescent participants was young people aged 12 to 17 years residing in non-temporary court-ordered foster care in New South Wales, Australia, where case supervision was provided by the statutory child welfare agency; who were not part of the baseline survey sample (i.e. baseline survey participants and non-participants); and whose placement address could be verified. Survey questionnaires were sent to the caregivers of 290 eligible participants residing at residential addresses with telephone contact, of which 145 were completed and returned (50% response rate).

### Caregiver Survey Questions on Intellectual and Speech-Language Difficulties

The survey caregiver questionnaires included questions pertaining to children’s ID, SLD and academic difficulties, which are listed in Table 1. For the present analysis of child survey data, index children were recorded as having ID if their caregiver answered ‘yes’ to Q1; and as having SLD if their caregiver answered ‘yes’ to Q3 and/or Q4. For the adolescent survey data, index adolescents were recorded as having ID if their caregiver answered ‘yes’ to any of questions Q2ii, Q2iii, Q2iv, Q2v or Q2viii (depending on the description); and as having SLD if they answered ‘yes’ to Q3 and/or Q6.

**Table 1:** Caregiver survey questions pertaining to children’s intellectual, speech-language, and academic difficulties

**Child survey questions:**	
*1. Does your child have an intellectual disability?*	
*2. If you answered yes, then what degree of intellectual disability does your child have? (Mild / Moderate / Severe / Profound / Not sure, or unknown)*	
*3. Does your child have a speech problem?*	
*4. Does your child attend speech therapy?*	
*5. If not, then has your child attended speech therapy previously?*	
*6. Is your child having difficulty learning to read?*	
**Adolescent survey questions**:	
*1. Does your child have learning difficulties?*	
*2. If so, then what type(s) of learning difficulties does he/she have?*	
*i. Not sure or don’t know*	
*ii. Borderline intellectual disability*	
*iii. Mild intellectual disability*	
*iv. Moderate, severe or profound intellectual disability*	
*v. Intellectual disability (don’t know the type or range)*	
*vi. Autism spectrum disorder, Asperger’s syndrome, or pervasive developmental disability*	
*vii. Specific learning disability or learning disorder (e.g. Reading Disorder)*	
*viii. Other learning difficulties (Describe*:	
*3. Does your child presently have a speech or language problem?*	
*4. If you answered Yes, please describe the problem*	
*5. If you answered No, has your child previously had a speech or language problem?*	
*6. Does your child presently receive speech or language therapy?*	
*7. If you answered no, have they previously received speech or language therapy?*	
*8. Compared to other children her age, does your child have reading difficulties?*	
*9. Compared to other children her age, does your child have arithmetic difficulties?*	

### Mental Health Measures

Child and adolescent mental health was measured prospectively using two series of caregiver-report checklists: the Child Behavior Checklist (CBCL) (Achenbach, 1991; Achenbach & Rescorla, 2001); and the Assessment Checklists for Children (ACC) and Adolescents (ACA) (Tarren-Sweeney, 2007; Tarren-Sweeney, 2013a). The reliability of foster carers’ reports of children’s difficulties remains somewhat uncertain, although there is evidence that in respect of children in long-term care, foster carers are at least as reliable as parents (Tarren-Sweeney et al., 2004). However, the evidence is less encouraging with regard to foster carers’ identification of adolescent difficulties in relatively new placements (Simmel et al., 2014).

#### CBCL

The CBCL measures common child mental health difficulties across eight empirically derived clinical subscales; two higher-order, broadband scales approximating spectrums of depressive/anxious symptoms (internalizing) and disruptive behavioural symptoms (externalizing); and a total problems score that provides a measure of global psychopathology. Additionally, the CBCL has six DSM-oriented scales derived through expert ratings of items’ conforming to DSM-IV-TR diagnostic criteria (Achenbach & Rescorla, 2001). CBCL scale score distributions are delineated into *normal*, *borderline clinical* and *clinical* ranges.

#### ACC / ACA

The ACC and ACA were developed for the present study to measure a range of mainly attachment- and trauma-related mental health difficulties manifested among children and young people in care, which are not adequately measured by standard rating instruments, such as the CBCL, and the Strengths and Difficulties Questionnaire (SDQ) (Goodman, 2001). Employing the CBCL and ACC/ACA in tandem thus provided comprehensive symptom coverage in the study.

The ACC is a 120-item carer-report checklist that measures behaviours, emotional states, traits, and social relatedness difficulties, as experienced by children in care aged 4 to 11 years, and related populations (Tarren-Sweeney, 2007). It has ten clinical sub-scales derived empirically through factor analysis, as well as two low self-esteem sub-scales derived using a different procedure. The clinical sub-scales are labelled: Sexual behaviour; Pseudomature interpersonal behaviour; Non-reciprocal interpersonal behaviour; Indiscriminate interpersonal behaviour; Insecure interpersonal behaviour; Anxious-distrustful; Abnormal pain response; Food maintenance; Self-injury; and Suicide discourse. The ACA is a 105-item carer-report mental health rating scale, measuring behaviours, emotional states, traits, and manners of relating to others, as manifested by adolescents residing in OOHC (Tarren-Sweeney, 2013a). In addition to a total clinical score, the ACA has seven clinical scales derived through factor analysis, that measure mostly attachment- and trauma-related symptomatology, labelled: Non-reciprocal Interpersonal Behaviour; Social Instability / Behavioural Dysregulation; Emotional Dysregulation / Distorted Social Cognition; Dissociation / Trauma Symptoms; Food Maintenance Behaviour; Sexual Behaviour Problems; and Suicide discourse.

For each ACC/ACA scale, cut-points define scores that indicate ‘possible’ and ‘probable’ clinical-level problems. Scores above the higher cut-points constitute *clinical* ranges that are highly predictive of psychiatric impairment (highly specific), while scores above the lower cut-point ranging up to and including the high cut-point constitute *elevated* ranges that retain high sensitivity for detecting psychiatric impairment. The ACC and ACA have good content, construct and criterion-related validity, as well as high internal reliability.

### Defining Mental Health Caseness and Case Complexity

In the present article, the term ‘cases’ refers to children who have clinically meaningful mental health difficulties requiring clinical intervention or support. Index children and adolescents were previously classified as cases for cluster analyses of CBCL and ACC/ACA scale scores, if they had one or more CBCL or ACC/ACA scale score in the clinical range (Tarren-Sweeney, 2013b; Tarren-Sweeney, 2021). For the present analyses, child and adolescent cases were further assigned to ‘borderline clinical’, ‘non-complex clinical’ and ‘complex clinical’ categories, based on their symptom profile severity and complexity identified in the aforementioned cluster analyses. While inattention is a core symptom of ADHD, impaired attention (and with it other executive functions) are also components of ID, FASD, traumatic brain injury and a range of other neurodevelopmental conditions. Therefore, for the present analyses, children and adolescents were not classified as cases if their only clinical range score was on the CBCL Attention Problems scale.

### Family Stress / Burden of Care Measure in the Adolescent Survey

The adolescent survey caregiver questionnaire included three scales from the Australian Adaptation of the Child Health Questionnaire (CHQ PF50) (Waters et al., 2000) that measure family stress and burden of care, named ‘Family activities’ (FA), ‘Emotional impact on caregiver’ (EI) and ‘Time impact on caregiver’ (TI). Raw scale mean scores are converted to scaled scores (transformed raw scores), with a possible range of 0–100, with lower scores indicating higher family stress and burden of care (Waters et al., 1999). The relevant GHQ items are listed in Table 2.

**Table 2:** Child Health Questionnaire items measuring family stress and burden of care in the adolescent survey

**‘Emotional impact on caregiver’ (EI) scale**	
*During the past 4 weeks*,* how much emotional worry or concern did each of the following cause you*:	
Your child’s physical health?	
Your child’s emotional well-being or behaviour?	
Your child’s attention or learning abilities?	
**‘Time impact on caregiver’ (TI) scale**	
*During the past 4 weeks*,* were you limited in the amount of time you had for your own personal needs because of*:	
Your child’s physical health?	
Your child’s emotional well-being or behaviour?	
Your child’s attention or learning abilities?	
**‘Impact on family activities’ (FA) scale**	
*During the past 4 weeks*,* how often has your child’s health or behaviour*:	
Limited the types of activities you could do as a family?	
Interrupted various everyday family activities (e.g. eating meals, watching TV)?	
Limited your ability as a family to “pick up and go” on a moment’s notice?	
Caused tension or conflict in your home?	
Been a source of disagreements or arguments in your family?	
Caused you to cancel or change plans (personal or work) at the last minute?	

## Results

The child survey sample (*N* = 347) had a mean age of 7.8 years, ranging from 4 to 11 years, with most (> 60%) being 6–8 years. Gender was evenly distributed (276 boys, 271 girls), and the numbers residing in foster and kinship care were 297 (86%) and 50 (14%), respectively. The adolescent survey sample (*N* = 230) included 85 young people recruited in the baseline survey, and 145 newly recruited subjects. Adolescents had a mean (median) age of 15.3 (15.0) years, ranging from 10.7 to 18.6 years. There were slightly more boys (*N* = 125) than girls (*N* = 105), while the numbers residing in foster and kinship care were 196 (85%) and 34.

(15%) respectively. Further details about the child and adolescent samples, including their maltreatment and OOHC histories are reported elsewhere (Tarren-Sweeney & Hazell, 2006; Tarren-Sweeney, 2018).

The proportions of children and adolescents with caregiver-reported ID were 22.5% (78/347) and 30.0% (69/230) respectively, while the proportions with reported SLD were 21.6% (75/347) and 15.7% (36/230) respectively. Around half (50/103) of children with ID or SLD were reported to have both types of difficulties, whereas only a third (26/79) of adolescents with ID or SLD had both.

Mean child CBCL and ACC mental health scores stratified by ID and SLD are listed in Table 3, and the equivalent adolescent CBCL and ACA score distributions are listed in Table 4. The study had sufficient power to identify statistically significant Cohen’s *d* effect sizes (group mean score differences expressed as a proportion of the aggregate standard deviation) above about 0.25 for the child data, and 0.28 for the adolescent data. There were sizeable numbers of statistically significant small (> 0.3), moderate (> 0.5) and large (> 0.8) effect sizes. Effect sizes for the two adolescent disability groups were generally larger than for the equivalent child groups. An interesting finding is that whereas the child ID group effect sizes were generally larger than those for the child SLD group, this pattern was reversed among the adolescent sample.

**Table 3:** Mean (SD) child (4–11 years) mental health scores stratified by caregiver-reported ID and SLD (*N* = 347)

	ID		Non-ID		d^b^	*p* ^c^		SLD		Non-SLD		d	*p*
	*N* = 78		*N* = 269					*N* = 75		*N* = 272			
**CBCL scales**													
Internalizing	10.7	(9.4)	8.4	(7.6)	0.28	*		10.6	(8.7)	8.5	(7.9)	0.27	*
Externalizing	20.4	(13.4)	16.2	(12.8)	0.32	*		19.3	(12.9)	16.6	(13.1)	0.21	
Anxious-depressed	4.9	(4.8)	4.0	(4.0)	0.22			4.8	(4.4)	4.1	(4.1)	0.18	
Withdrawn-depressed	3.2	(3.3)	2.2	(2.5)	0.36	**		3.1	(3.2)	2.3	(2.6)	0.28	*
Somatic complaints	2.6	(3.2)	2.1	(2.7)	0.14			2.7	(3.0)	2.1	(2.8)	0.22	
Social problems	8.2	(4.3)	4.9	(3.9)	0.76	***		7.8	(4.1)	5.1	(4.1)	0.64	***
Thought problems	5.8	(4.8)	3.7	(4.1)	0.49	***		5.2	(4.7)	3.9	(4.2)	0.28	*
Attention problems	10.9	(5.1)	6.6	(4.8)	0.83	***		10.0	(5.5)	6.9	(4.9)	0.60	***
Rule-breaking behaviour	6.4	(5.3)	5.3	(5.0)	0.22			5.8	(5.0)	5.5	(5.1)	0.05	
Aggressive behaviour	14.0	(8.9)	10.9	(8.4)	0.36	**		13.5	(8.4)	11.1	(8.6)	0.29	*
**ACC scales**													
Sexual behaviour problems	1.6	(3.2)	1.2	(2.6)	0.18			1.0	(2.0)	1.3	(2.9)	0.11	
Pseudomature	3.1	(3.1)	3.3	(3.7)	0.07			2.9	(3.1)	3.4	(3.7)	0.12	
Non-reciprocal	6.2	(5.3)	4.0	(4.4)	0.47	***		5.1	(5.0)	4.3	(4.6)	0.18	
Indiscriminate	8.3	(4.1)	5.8	(4.0)	0.61	***		7.4	(4.2)	6.1	(4.1)	0.31	*
Insecure	6.4	(6.0)	4.8	(4.8)	0.31	*		5.6	(5.7)	5.0	(5.0)	0.11	
Anxious-distrustful	2.9	(3.0)	2.2	(3.1)	0.23			2.9	(3.0)	2.2	(3.1)	0.22	
Abnormal pain response	1.6	(2.3)	1.0	(1.7)	0.32	*		1.5	(2.4)	1.1	(1.7)	0.21	
Food maintenance	1.6	(2.4)	1.0	(1.8)	0.32	*		1.5	(2.3)	1.0	(1.9)	0.22	
Self-injury	2.4	(4.3)	1.0	(2.1)	0.50	***		2.4	(4.5)	1.0	(2.1)	0.50	***
Suicide discourse	0.2	(0.9)	0.3	(1.1)	0.05			0.1	(0.5)	0.3	(1.2)	0.16	

^a^ Cohen’s *d*

^b^ Two-tailed significance level (t-test, unequal variance): ns (not significant); * *P* ≤.05; ** *P* ≤.01; *** *P* ≤.001

**Table 4:** Mean (SD) adolescent (12–18 years) mental health scores stratified by caregiver-reported ID and SLD (*N* = 230)

	ID		Non-ID		d^a^	*p* ^b^		SLD		Non-SLD		d	*p*
	*N* = 69		*N* = 161					*N* = 36		*N* = 194			
**CBCL scales**													
Internalizing	10.9	(8.3)	7.4	(7.2)	0.45	**		13.3	(9.8)	7.6	(6.9)	0.74	***
Externalizing	18.9	(12.8)	12.1	(11.7)	0.55	***		20.1	(13.8)	13.0	(11.9)	0.57	**
Anxious-depressed	5.0	(4.8)	3.1	(3.4)	0.48	***		5.8	(5.7)	3.3	(3.5)	0.63	***
Withdrawn-depressed	3.6	(3.1)	2.6	(2.8)	0.38	**		4.8	(2.9)	2.5	(2.8)	0.76	***
Somatic complaints	2.2	(2.3)	1.8	(2.4)	0.18			2.8	(3.0)	1.8	(2.3)	0.42	*
Social problems	6.6	(4.7)	3.2	(3.8)	0.76	***		7.7	(4.3)	3.6	(4.0)	0.95	***
Thought problems	5.3	(5.0)	2.5	(3.1)	0.70	***		6.7	(5.3)	2.8	(3.4)	1.00	***
Attention problems	10.6	(4.9)	5.9	(4.9)	0.89	***		11.0	(5.2)	6.7	(5.1)	0.81	***
Rule-breaking behaviour	7.3	(6.1)	4.6	(5.0)	0.49	***		7.5	(6.4)	5.0	(5.2)	0.45	**
Aggressive behaviour	11.7	(8.1)	7.5	(7.4)	0.53	***		12.7	(8.8)	8.0	(7.4)	0.59	***
**ACA scales**													
Non-reciprocal	5.1	(4.2)	3.0	(3.5)	0.54	***		5.8	(4.6)	3.2	(3.6)	0.67	***
SI/BD ^c^	9.5	(8.7)	6.7	(7.4)	0.35	*		10.3	(9.3)	7.0	(7.5)	0.42	*
ED/DSC ^d^	5.0	(5.7)	3.6	(4.8)	0.29	*		7.0	(7.0)	3.4	(4.5)	0.71	***
Dissociation / Trauma sym.	1.6	(2.2)	0.6	(1.5)	0.54	***		2.4	(2.9)	0.6	(1.3)	1.00	***
Food maintenance	2.1	(3.3)	1.4	(2.6)	0.25			2.6	(3.9)	1.5	(2.6)	0.39	*
Sexual behaviour problems	0.9	(2.4)	0.2	(0.9)	0.40	**		0.6	(2.1)	0.4	(1.4)	0.10	
Suicide discourse	0.5	(1.8)	0.1	(0.7)	0.30	**		0.3	(1.8)	0.2	(1.0)	0.08	

^a^ Cohen’s *d*

^b^ Two-tailed significance level (t-test, unequal variance): ns (not significant); * *P* ≤.05; ** *P* ≤.01; *** *P* ≤.001

^c^ Social instability / Behavioural dysregulation

^d^ Emotional dysregulation / Distorted social cognition

The proportions of children and adolescents identified as mental health cases were 67.1% (233/347) and 60.4% (139/230) respectively. Contingency Table (2 × 2) of mental health caseness by ID, current SLD and any history of SLD (i.e. current or prior SLD) are listed with odds ratios in Table 5. Odds ratios range from 1.7 to 2.7 among the child sample, and from 2.8 to 6.5 among the adolescent sample (all statistically significant), showing that children and adolescents with ID and/or SLD are consistently more likely to have clinical-level mental health difficulties than other children in care. Indeed, large majorities of children and adolescents with ID (82.1% and 81.1% respectively), and with SLD (77.3% and 88.9% respectively) had co-occurring mental health difficulties. Among the aggregate samples, 23.9% (83/347) of children and 28.7% (66/230) of adolescents were identified with inter-developmental impairment – defined as having ID *and/or* SLD, as well as mental health caseness.

**Table 5:** Rates of mental health caseness and complexity by caregiver-reported developmental difficulties

	Dichotomous caseness ^a^ (2 × 2 groups)		Case complexity (4 × 2 groups)			
CHILDREN (N = 347)	*Cases* *(N = 233)*	*Non-cases* *(N = 114)*	*Complex clinical* *(N = 70)*	*Non-complex clinical (N = 119)*	*Borderline clinical* *(N = 44)*	*Non-cases (N = 114)*
***ID (%)***	OR = 2.7 (1.5–5.0) ^b^		Chi2 = 20.8, *p* < .001			
Yes, *N* = 78 (22.5%)	64 (82.1%)	14 (17.9%)	21 (26.9%)	39 (50.0%)	4 (5.1%)	14 (18.0%)
No, *N* = 269 (77.5%)	169 (62.8%)	100 (37.2%)	49 (18.2%)	80 (29.7%)	40 (14.9%)	100 (37.2%)
***Current SLD*** ^***c***^ ***(%)***	OR = 1.9 (1.0–3.8)		Chi2 = 6.0, *p* = .11			
Yes, *N* = 75 (21.6%)	58 (77.3%)	17 (22.7%)	18 (24.0%)	32 (42.7%)	8 (10.7%)	17 (22.7%)
No, *N* = 272 (78.4%)	175 (64.3%)	97 (35.7%)	52 (19.1%)	87 (32.0%)	36 (13.2%)	97 (35.7%)
***History of SLD*** ^***d***^ ***(%)***	OR = 1.7 (1.1–2.8)		Chi2 = 13.7, *p* = .003			
Yes, *N* = 119 (34.3%)	89 (74.8%)	30 (25.2%)	26 (21.9%)	54 (45.4%)	9 (7.6%)	30 (25.2%)
No, *N* = 228 (65.7%)	144 (63.2%)	84 (36.8%)	44 (19.3%)	65 (28.5%)	35 (15.4%)	84 (36.8%)

*ADOLESCENTS (N = 230)*	*Cases* *(N = 139)*	*Non-cases* *(N = 91)*	*Complex clinical* *(N = 35)*	*Non-complex clinical* *(N = 69)*	*Borderline clinical* (*N = 35)*	*Non-cases (N = 91)*
*ID (%)*	*OR = 4.0 (2.1–7.9)* ^b^		*Chi2 = 20.8, p < .001*			
Yes, *N* = 69 (30.0%)	56 (81.1%)	13 (18.8%)	15 (21.7%)	31 (44.9%)	10 (14.5%)	13 (18.8%)
No, *N* = 161 (70.0%)	83 (51.6%)	78 (48.5%)	20 (12.4%)	38 (23.6%)	25 (15.5%)	78 (48.5%)
***Current SLD*** ***(%)***	OR = 6.5 (2.3–18.3)		Chi2 = 16.2, *p* = .001			
Yes, *N* = 36 (15.7%)	32 (88.9%)	4 (11.1%)	10 (27.8%)	16 (44.4%)	6 (16.7%)	4 (11.1%)
No, *N* = 194 (84.3%)	107 (55.1%)	87 (44.8%)	25 (12.9%)	53 (27.3%)	29 (14.9%)	87 (44.8%)
***History of SLD*** ***(%)***	OR = 2.8 (1.4–5.3)		Chi2 = 13.1, *p* = .005			
Yes, *N* = 64 (27.8%)	49 (76.6%)	15 (23.4%)	11 (17.2%)	29 (45.3%)	9 (14.1%)	15 (23.4%)
No, *N* = 166 (72.2%)	90 (54.2%)	76 (45.8%)	24 (14.5%)	40 (24.1%)	26 (15.7%)	76 (45.8%)

^a^ Non-cases = Normative mental health; Cases = Clinical or ‘borderline clinical’ difficulties

^b^ Odds Ratio (95% confidence interval)

^c^ Reported current speech-language difficulties

^d^ Reported history of any speech-language difficulties, including current difficulties

Among the child and adolescent cases, 18.9% (44/233) and 25.2% (35/139) respectively were borderline clinical cases; 51.1% (119/233) and 49.6% (69/139) respectively were non-complex clinical cases; and 30.0% (70/233) and 25.2% (35/139) respectively were complex clinical cases. Contingency Table (4 × 2) of mental health case complexity (four groups) by ID, current SLD, and any history of SLD are listed in Table 5. All of the Chi2 values are statistically significant, except for child SLD. These latter analyses demonstrate that the relationship between developmental status and mental health status extends to case complexity and severity.

Associations between the caregiver CHQ scale scores and adolescents’ mental health case complexity, ID, SLD, gender, age and care history, are listed in Table 6. Australian normative mean scores are included in the table for comparison. Caregiver ‘burden of care’ scores were strongly associated with adolescents’ mental health case complexity, ID and SLD, but were not associated with their gender, age, or care histories (‘age at entry into care’ and ‘number of placements in care’).

**Table 6:** Associations between CHQ family stress / burden of care scores ^a^ and adolescents’ mental health case complexity, ID, SLD, gender, age and care history (*N* = 230)

	Emotional impact on caregiver	Time impact on caregiver	Impact on family activities
Mental health case complexity	*F* = 54.6, *p* < .0001	*F* = 32.8, *p* < .0001	*F* = 76.7, *p* < .0001
*Non-cases (n = 89)*	*M* = 90.2	*M* = 93.3	*M* = 91.1
*Borderline clinical (n = 36)*	*M* = 70.6	*M* = 79.3	*M* = 75.5
*Non-complex clinical (n = 70)*	*M* = 61.7	*M* = 69.1	*M* = 57.5
*Complex clinical (n = 35)*	*M* = 48.3	*M* = 58.4	*M* = 33.3
Reported ID	*t* = 5.56, *p* < .0001	*t* = 4.29, *p* < .0001	*t* = 4.40, *p* < .0001
*No (n = 161)*	*M* = 77.5	*M* = 82.7	*M* = 74.7
*Yes (n = 69)*	*M* = 59.3	*M* = 68.4	*M* = 56.9
Reported SLD	*t* = 5.60, *p* < .0001	*t* = 5.16, *p* < .0001	*t* = 3.87, *p* = .0001
*No (n = 194)*	*M* = 75.7	*M* = 81.7	*M* = 72.5
*Yes (n = 36)*	*M* = 52.5	*M* = 60.5	*M* = 52.7
Gender	*t* = 0.23, *p* = .82	*t* = 0.86, *p* = .39	*t* = 1.11 *p* = .29
*Boys*	*M* = 72.4	*M* = 79.6	*M* = 67.5
*Girls*	*M* = 71.7	*M* = 76.9	*M* = 71.6
Age	*r* = .09, *p* = .16	*r* = .10, *p* = .12	*r* = .07, *p* = .92
Age at entry into care	*r* = .07, *p* = .37	*r* = .05, *p* = .53	*r* = .06, *p* = .43
Number of care placements	*r* = −.09, *p* = .39	*r* = −.05, *p* = .67	*r* = −.08, *p* = .49
*Australian normative mean scores* ^*b*^	*M = 78–80*	*M = 91–94*	*M = 85–86*

^a^ CHQ scores scaled from 0-100, lower scores indicate greater impact. Community mean scores for this age range

^b^ Comparative mean normative scores for Australian population. Separate norms for boys and girls, and for 13–15 and 16–18 year-olds (Waters et al., 2000)

Linear regressions and regression diagnostics were performed in Stata (StataCorp, 2019) to estimate the extent to which mental health case complexity and reported intellectual and language difficulties independently predict caregiver ‘burden of care’. The demographic and care history variables were excluded from the regression models because they were not associated with burden of care scores. Data and regressions were checked for normality, homoscedasticity, and multicollinearity. The EI, TI and FA scores were highly skewed in favour of higher scores, and were thus transformed to natural log with zero skew using the Stata ‘lnskew0’ function. For each of the regression models, residuals were homoscedastic (Breusch-Pagan / Cook-Weisberg test), and the variables showed low multi-collinearity tolerance (1/VIF ranged from 1.13 to 1.26). Three linear regression models, predicting EI, TI and FA respectively by adolescents’ mental health case complexity, ID and SLD (*N* = 230) are outlined in Table 7. The models predicted relatively large proportions of the EI, TI and FA score variances, as indicated by the adjusted *R* squared statistic (0.45, 0.31 and 0.49 respectively). While all three variables independently predicted the ‘emotional impact on caregiver’, mental health case complexity was a much stronger predictor (larger independent effect size, as indicated by standardized beta coefficients) than were ID or SLD. Mental health case complexity strongly predicted ‘time impact on caregiver’, while SLD had a much smaller effect, and ID was not significant. Mental health case complexity also strongly predicted ‘impact on family activities’, while neither ID nor SLD predicted FA.

**Table 7:** Linear regression models: prediction of ‘emotional impact on caregiver’, ‘time impact on caregiver’ and ‘impact on family activities’ by adolescents’ mental health case complexity, reported ID and SLD (*N* = 230)

	Adjusted *R* squared	Correlation	Partial correlation	βeta	*p* value
**EI Model**: ***Emotional impact on caregiver***	0.45				< 0.0001
Mental health case complexity		− 0.65	− 0.60	− 0.58	< 0.0001
Reported ID		− 0.35	− 0.15	− 0.12	0.03
Reported SLD		− 0.32	− 0.15	− 0.12	0.02
**TI Model**: ***Time impact on caregiver***	0.31				< 0.0001
Mental health case complexity		− 0.55	− 0.50	− 0.50	< 0.0001
Reported ID		− 0.25	− 0.06	− 0.05	0.40
Reported SLD		− 0.27	− 0.14	− 0.13	0.04
**FA Model**: ***Impact on family activities***	0.49				< 0.0001
Mental health case complexity		− 0.71	− 0.67	− 0.69	< 0.0001
Reported ID		− 0.27	− 0.06	− 0.05	0.38
Reported SLD		− 0.21	− 0.02	− 0.02	0.72

## Discussion

The present study findings add to our limited understanding of the relationships between different types of developmental impairment among children and adolescents in foster and kinship care – including the prevalence and impact of inter-developmental impairment. While mental health case rates were high among the aggregate child (67%) and adolescent (60%) samples, those with caregiver-reported ID and/or SLD were considerably more likely to be mental health cases compared to those without ID or SLD, with odds ratios ranging from 1.9 to 6.5. The most important finding was that roughly one quarter of children and adolescents in foster care are estimated to manifest inter-developmental impairment – defined as having ID *and/or* SLD, as well as mental health caseness. Looking beyond dichotomous caseness, we found strong associations between developmental difficulties and mental health case complexity. For example, whereas 12.4% of adolescent non-cases had reported ID, 42.9% of adolescent complex cases had reported ID. The study findings also provide some insight into the relative toll that different types of developmental impairment have on caregiving and family life. The present analyses suggest that adolescent mental health difficulties have considerably greater negative impacts on caregivers’ feelings and time, as well as on family activities, than do adolescents’ cognitive or language difficulties.

### What Might Account for a High Rate of Inter-Developmental Impairment among Children in OOHC?

Children in OOHC constitute a relatively small sub-set of maltreated children who have generally experienced more severe, more chronic, more pervasive and more diverse maltreatment,as well as other social, physical and biological adversity. Early exposure to such severe adversities incurs profound and long-standing effects on children’s psychological / neurological development – accounting for impairments in cognitive, language, social, emotional and behavioural functioning. A range of psychological and neuro-biological processes in early childhood that are critical to human social functioning are impaired through early and prolonged exposure to traumatic abuse, and by the absence of nurturing, sensitive care (Charlotte et al., 2017; Young-Southward et al., 2020). In jurisdictions where children predominantly enter long-term care following severe and persistent maltreatment, a child’s age at entry into care approximates their length of post-birth exposure to chronic and severe maltreatment. Furthermore, a child’s ‘age at entry into care’ strongly predicts their subsequent mental health difficulties – with entry at younger age being protective (Burge, 2007; Hukkanen et al., 1999; Tarren-Sweeney, 2008). This is consistent with cumulative trauma exposure models (Charlotte et al., 2017), neuroscience, and attachment theory. Cumulative trauma exposure also accounts for increasing symptom complexity in both childhood and adulthood (Cloitre et al., 2009). However, trauma exposure does not solely explain the complexity of inter-developmental impairment manifested by maltreated children placed in OOHC. While maltreatment includes child abuse that is psychologically and/or physically traumatic, it also includes neglect. Among child welfare scholars, policy makers and practitioners there is a growing tendency to frame the biopsychosocial development of children in OOHC exclusively through a *developmental trauma* lens, especially with respect to their mental health. We have noticed a parallel trend among our clinical colleagues to formulate complex symptomatology under a cloak of undifferentiated developmental trauma. ICD-11 Complex Post-traumatic Stress Disorder (C-PTSD) represents the latest attempt to conceptualise complex trauma symptoms as a unitary disorder (World Health Organization, 2020). However, a recent analysis of the present adolescent sample’s mental health scores identified only three young people (2% of cases) with symptoms matching C-PTSD criteria (Lawless & Tarren-Sweeney, 2022).

One implication of formulating too narrowly within a developmental trauma frame, is that we can lost sight of the effects that profound deprivation of physical, emotional and social care have on infant and early childhood development. The English and Romanian Adoptees (ERA) study provided solid evidence that profound social deprivation in infancy impairs and/or delays development of language, attention, cognition, and social / relational capacity – involving multiple biopsychosocial mechanisms (including impaired executive function, theory of mind, and attachment development) (Colvert et al., 2008; Kreppner et al., 2001). Similarly, a study of UK adolescents in OOHC with complex or high psychopathology identified a link between disordered attachment, social cognitive deficits and mental health difficulties (Kay & Green, 2016). While most of the Romanian adoptees developed meaningful attachments and showed cognitive and language recovery after being placed with their adoptive families, those exposed to more than six months of profoundly depriving institutional care showed persistently high rates of inattention-overactivity, disinhibited social engagement, and autism spectrum disorder symptoms into young adulthood (Sonuga-Barke et al., 2017).

In addition to being psychologically traumatic, physical abuse also harms children’s psychological and sensory development via traumatic brain injury (TBI). One recent study suggests that TBIs are relatively common among children and adolescents in OOHC, and are largely undetected (Cusimano et al., 2021). Additional developmental risk exposures include maladaptive social learning conditions; high genetic load for mental illness (high rates of parental psychopathology) (Green et al., 2019); and high exposure to pre-natal adversity affecting foetal brain development (maternal alcohol and substance use, and high maternal stress in pregnancy) (Marcellus & Badry, 2023; Vig et al., 2005). Complex risk exposures in turn contribute to a complex latent vulnerability phenotype (McCrory & Viding, 2015), that likely moderates the risk of emerging mental illness in adolescence. Finally, children in OOHC may experience high rates of specific types of emotional and behavioural difficulties that are more commonly manifested by children with developmental disabilities or specific syndromes (Buckley et al., 2020), on account of those developmental disabilities being more prevalent among children in care. For example, in the present study reported intellectual disability was more strongly associated with pica-type behaviours, food maintenance behaviours (storing, hoarding, gorging), encopresis, self-injury, inattention, and disinhibited social engagement – in comparison to other mental health difficulties (Tarren-Sweeney, 2006).

### Study Limitation

While the survey questionnaire included robust and reliable mental health measures, children’s cognitive and language abilities were not formally measured. Ascertaining ID and SLD status from caregivers is likely to be somewhat inaccurate, given that: many children’s cognitive and language abilities may not have been formally assessed; assessment results may not be routinely communicated to foster carers; and some children had only been with their current caregivers for a short time. Nevertheless, at the time of the study NSW children with suspected ID were routinely assessed prior to starting school, and children with mild and moderate ID were routinely identified for special education.

### Implications for Clinical Formulation

The present study findings emphasize the need for comprehensive mental health and developmental assessments of children in out-of-home care that provide sufficient information to formulate complex inter-developmental impairment. The findings also highlight a need for more specialized clinical guidance and training on the intersections between complex maltreatment-related psychopathology and neurodevelopmental syndromes. Understanding inter-developmental impairment requires transdisciplinary knowledge and training that does not sit neatly within traditional clinical or developmental disciplines (child and adolescent psychiatry, clinical psychology, developmental paediatrics, special education, etc.). Finally, the present findings point to a need for integrated treatment and clinical management that simultaneously addresses the different components of inter-developmental impairment.
